# Supplementary material for: A 69 kbp Deletion at the Berry Color Locus Is Responsible for Berry Color Recovery in Vitis vinifera L. Cultivar ‘Riesling Rot’
Source: Int J Mol Sci. 2022 Mar 28;23(7):3708. doi: 10.3390/ijms23073708 (PMC8998622; doi:10.3390/ijms23073708)
Supplement: Supplementary file 1 [file ijms-23-03708-s001.zip › ijms-1639190 - sup revised/Supplemental Figures.pdf]

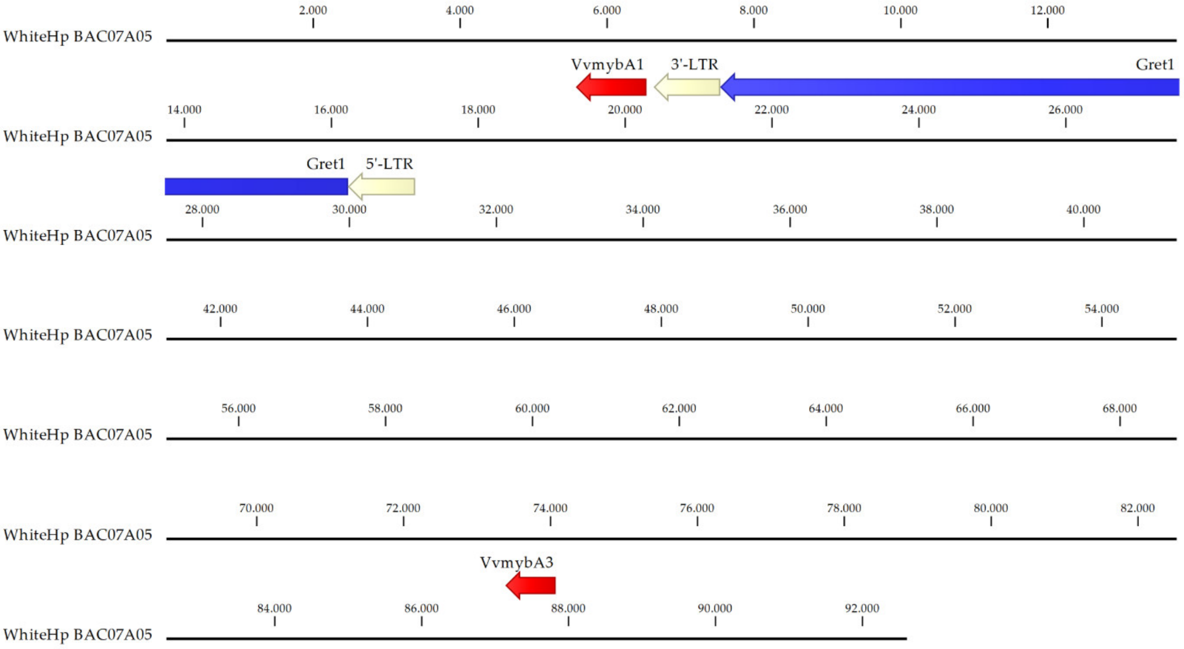

Figure S1. Schematic overview of the white haplophase of 'Riesling Rot' based on BAC clone sequencing. The positions of *VvmybA1*, *Gret1* and *VvmybA3* are highlighted.

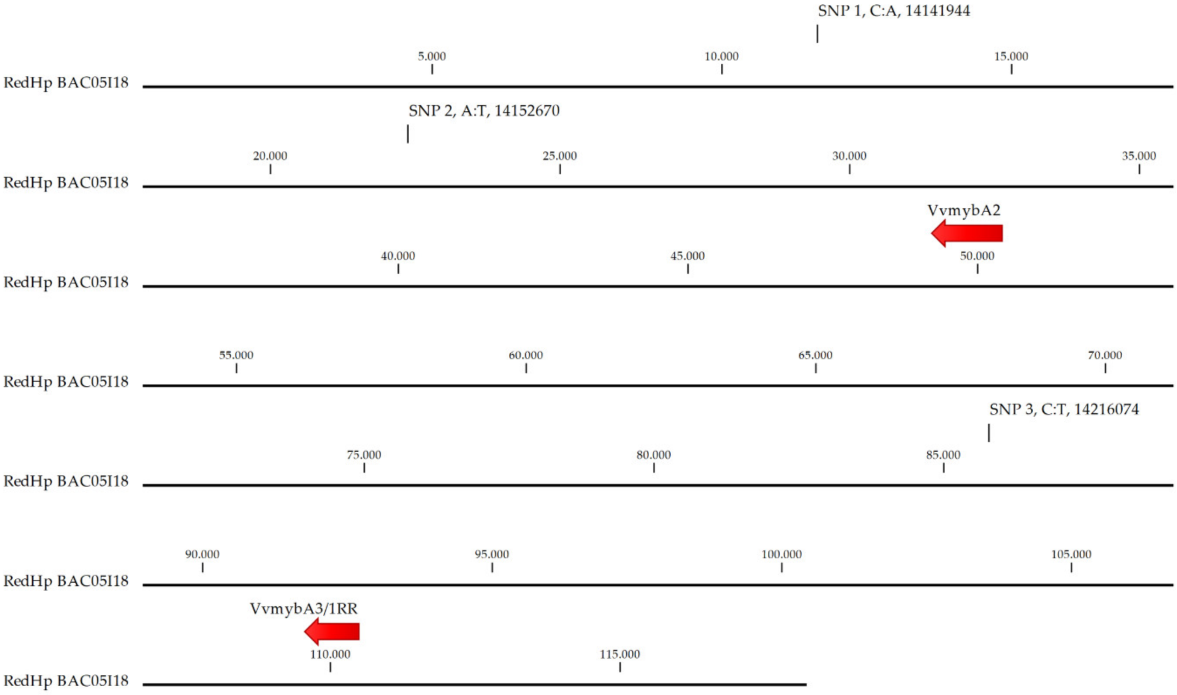

Figure S2. Schematic overview of the red (mutated) haplophase of 'Riesling Rot' based on BAC clone sequencing. The positions of *VvmybA2* and *VvmybA3/1RR* are highlighted. SNP positions are given relative to chromosome 2 of the PN40024 12x reference sequence (http://ensembl.gramene.org/Vitis_vinifera/Info/Index).

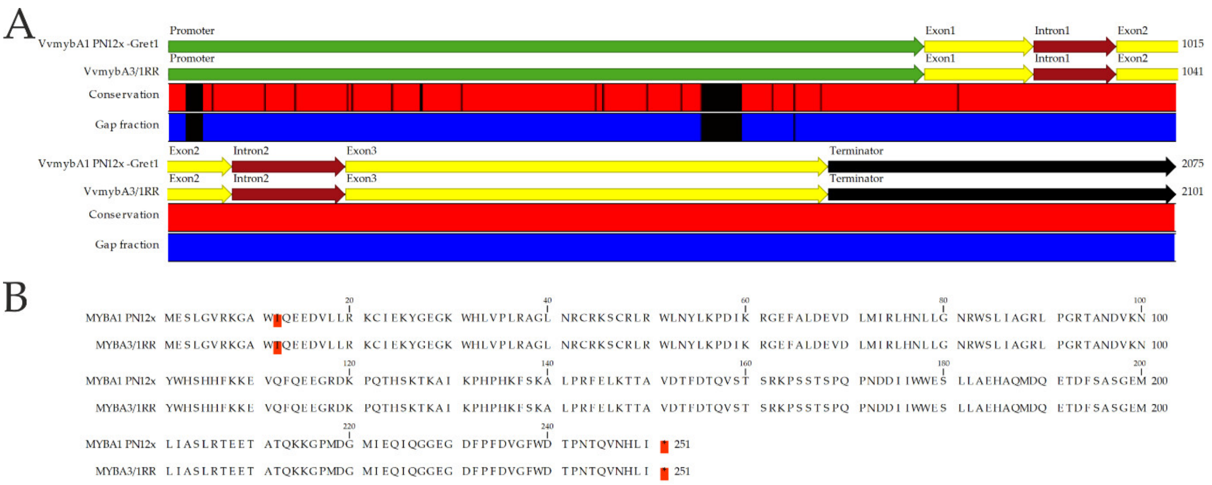

Figure S3. Alignments of the *VvmybA3/1RR* gene variant of 'Riesling Rot' and *VvmybA1* of the PN40024 (12x) reference genome sequence (VIT_02s0033g00410). (**A**) Sequence alignment with promoter and terminator region. To enhance comparability of promoter regions *Gret1* was manually deleted from the reference sequence. (**B**) Amino acid sequence alignment. The difference and stop codons are highlighted in red.

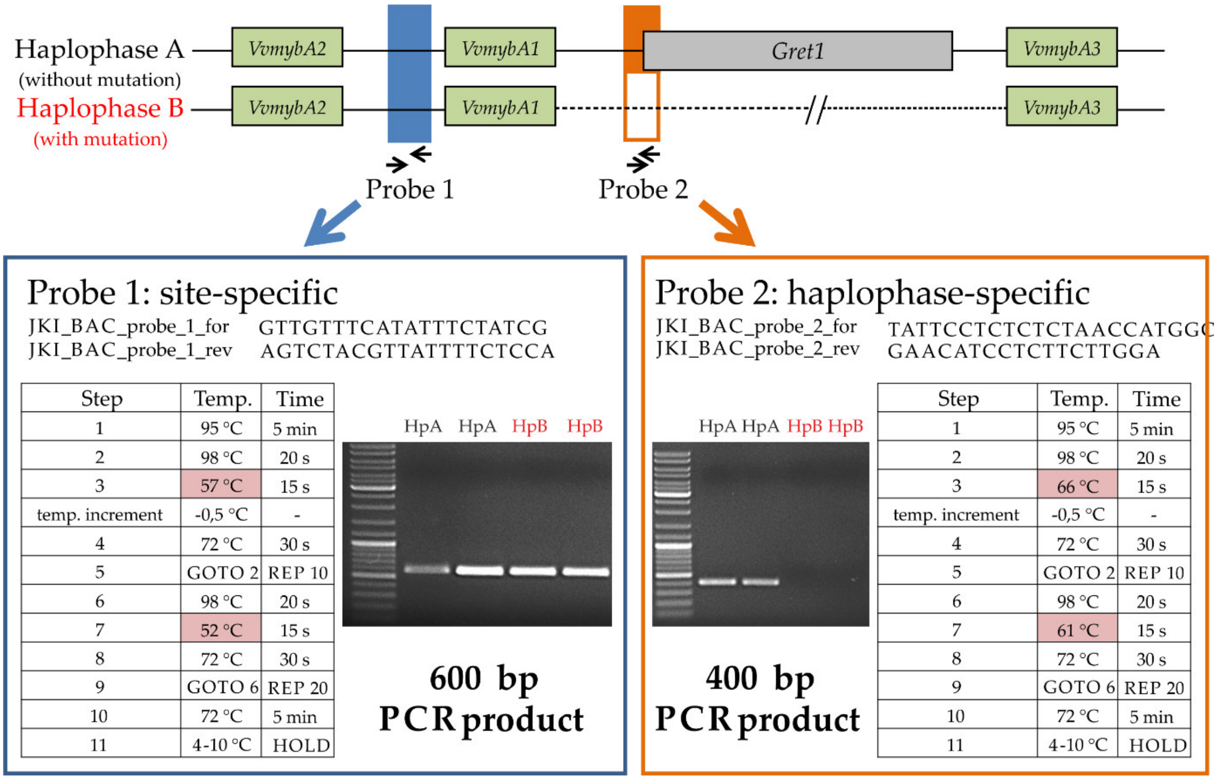

Figure S4. BAC library screening scheme performed at Clemson University's Genomics Institute. Probe 1 was designed to select BACs with a grapevine berry color locus insert, while Probe 2 was used specifically to distinguish between the white haplophase (A) and mutated red haplophase (B). The Test-PCR was performed using the KAPA HiFi Hot Start PCR Kit from Peqlab, Erlangen, Germany in the touch-down variant on the basis of genomic DNA of homozygous selfings of 'Riesling Rot' (RRs1, RRs3, RRs10 and RRs11) to differentiate the haplophases.
